# Resistance to Boscalid, Fluopyram and Fluxapyroxad in *Blumeriella jaapii* from Michigan (U.S.A.): Molecular Characterization and Assessment of Practical Resistance in Commercial Cherry Orchards

**DOI:** 10.3390/microorganisms9112198

**Published:** 2021-10-21

**Authors:** Jacqueline Gleason, Jingyu Peng, Tyre J. Proffer, Suzanne M. Slack, Cory A. Outwater, Nikki L. Rothwell, George W. Sundin

**Affiliations:** 1 Department of Plant, Soil, and Microbial Sciences, Michigan State University, E. Lansing, MI 48824, USA; gleaso97@msu.edu (J.G.); pengjin2@msu.edu (J.P.); proffer@msu.edu (T.J.P.); slacksuz@msu.edu (S.M.S.); outwate7@msu.edu (C.A.O.); 2 Northwest Michigan Horticultural Research Center, Traverse City, MI 49684, USA; rothwel3@msu.edu

**Keywords:** cherry leaf spot, succinate dehydrogenase inhibitor, *SdhB*, *SdhC*

## Abstract

Management of cherry leaf spot disease, caused by the fungus *Blumeriella jaapii*, with succinate dehydrogenase inhibitor (SDHI) fungicides has been ongoing in Michigan tart cherry orchards for the past 17 years. After boscalid-resistant *B. jaapii* were first isolated from commercial orchards in 2010, premixes of SDHI fungicides fluopyram or fluxapyroxad with a quinone outside inhibitor were registered in 2012. Here, we report widespread resistance to fluopyram (Fluo^R^), fluxapyroxad (Flux^R^), and boscalid (Bosc^R^) in commercial orchard populations of *B. jaapii* in Michigan from surveys conducted between 2016 and 2019. A total of 26% of 1610 isolates from the 2016–2017 surveys exhibited the fully-resistant Bosc^R^ Fluo^R^ Flux^R^ phenotype and only 7% were sensitive to all three SDHIs. Practical resistance to fluopyram and fluxapyroxad was detected in 29 of 35 and 14 of 35 commercial tart cherry orchards, respectively, in surveys conducted in 2018 and 2019. Sequencing of the SdhB, SdhC, and SdhD target genes from 22 isolates with varying resistance phenotypes showed that Bosc^S^ Fluo^R^ Flux^S^ isolates harbored either an I262V substitution in SdhB or an S84L substitution in SdhC. Bosc^R^ Fluo^R^ Flux^R^ isolates harbored an N86S substitution in SdhC, or contained the N86S substitution with the additional I262V substitution in SdhB. One Bosc^R^ Fluo^R^ Flux^R^ isolate contained both the I262V substitution in SdhB and the S84L substitution in SdhC. These mutational analyses suggest that Bosc^R^ Fluo^R^ Flux^R^ isolates evolved from fully sensitive Bosc^S^, Fluo^S^, Flux^S^ isolates in the population and not from boscalid-resistant isolates that were prevalent in the 2010–2012 time period.

## 1. Introduction

Cherry leaf spot (CLS), caused by the fungus *Blumeriella jaapii* (Rehm) Arx, is a significant disease problem on sour/tart cherry (*Prunus cerasus* L.). Ascospores of *B. jaapii* are produced in apothecia on overwintered leaves on the orchard floor. The ascospores are typically released near petal fall and infect leaves via the stomata. In years with moderately warm and wet weather during bloom, initial infections can occur on bract leaves adjacent to flowers; this situation commonly results in CLS epidemics that can have a major negative impact on fruit ripening. On untreated tart cherry trees of the highly-susceptible cultivar ‘Montmorency’ (~95% of the tart cherry industry in Michigan, USA), the incidence of CLS can increase from ~5% to ~100% within one month [1]. As leaves accumulate CLS lesions, they become chlorotic and abscise; CLS-mediated defoliation typically follows initial leaf infection by about two to four weeks [1]. With CLS disease in Michigan, in situations where defoliation levels of >50% occur prior to September, affected trees are at significant risk of winter injury and possible tree death [1].

*Blumeriella jaapii* is a prolific sporulator on ‘Montmorency’, and masses of conidia, visible to the naked eye, are formed on the abaxial leaf surface in acervuli. The conidia are readily spread by rain and wind causing secondary infections also via the stomata, making management of CLS extremely difficult under favorable weather conditions. Because of the high susceptibility of ‘Montmorency’, CLS management is entirely dependent upon the use of fungicides, with six to eight full cover applications used in a typical season. Broad-spectrum fungicides including chlorothalonil, captan, and coppers are effective in CLS control; however, growers have historically favored single-site fungicides that are either systemic or translaminar because these fungicides can control other diseases including powdery mildew (*Podosphaera clandestina* (Wallr.:Fr.) Lev.) and brown rot (*Monilinia fructicola* (G.Winter) Honey), and can be used with longer interval times between applications.

The evolution of fungicide resistance in populations of *B. jaapii* in Michigan has been an ongoing problem over the past 15–20 years. Resistance to the sterol demethylation inhibitor fungicide class emerged in the mid-2000s [2], and resulted from overexpression of the target *CYP51* gene due to insertion of an outwardly-directed promoter sequence carried by a transposable element [3]. Resistance to the succinate dehydrogenase inhibitor (SDHI) boscalid, a component of the premix fungicide Pristine (BASF Corporation; Research Triangle Park, NC) was first detected in 2010–2011 [4]. Frequent use of Pristine at this time in tart cherry orchards in Michigan quickly led to practical resistance in most commercial orchards in the state. These boscalid-resistant *B. jaapii* strains possessed the H260R mutation in the *SdhB* target gene (B-H260R) [4]; mutation of this conserved histidine residue to either arginine or tyrosine is very common among other boscalid-resistant fungi [5,6,7].

The SDH complex is encoded by four genes (*SdhA*, *SdhB*, *SdhC*, and *SdhD*) and comprises a critical component of aerobic respiration in fungal mitochondria [8]. Structural analyses of these proteins have shown that a complex of the *SdhB*, *SdhC*, and *SdhD* proteins generates a ubiquinone-binding pocket [6]. This pocket is targeted by most fungicides of the SDHI class, and mutations that alter the structure of the pocket can result in SDHI fungicide resistance [5]. However, because of the variety of chemical structures of SDHI fungicides discovered to date, mutations such as B-H277Y and B-H277R that changed the structure of the ubiquinone-binding pocket and prevented boscalid from targeting the SDH complex did not necessarily result in cross resistance to other SDHI fungicides such as fluopyram [9]. Similarly, we found that the boscalid-resistant *B. jaapii* strains isolated in Michigan were still susceptible to other SDHI fungicides, including fluopyram and fluxapyroxad [4], and so Pristine was quickly replaced in CLS disease control programs by the fungicides Luna Sensation (Bayer CropScience; St. Louis, MO, USA), which is a premix of fluopyram and the quinone outside inhibitor (QoI) trifloxystrobin, and Merivon (BASF), which is a premix of fluxapyroxad and the QoI pyraclostrobin. Both of these fungicides were registered for CLS disease control in 2012.

Reports of resistance to fluopyram and fluxapyroxad in fungi such as *Alternaria alternata, Botrytis cinerea*, and *Sclerotinia homoeocarpa* have been appearing in recent years [10,11,12,13,14,15,16]. Several *Sdh* gene mutations have been identified and shown to be correlated with fluopyram and/or fluxapyroxad resistance including C-G91R and C-G150R with fluxapyroxad resistance in *S. homoeocarpa* [15], B-P225F and B-N230I with fluopyram and fluxapyroxad resistance in *B. cinerea* [13,14], B-I229V in *SdhB* from a single fluopyram-resistant field isolate of *Stagonosporopsis citrulli* [17], and B-I280V in *SdhB* from fluopyram-resistant *Corynespora cassiicola* [18]. The location for both of the isoleucine to valine mutants discovered in *SdhB* is only two amino acids away from the conserved His residue, where mutation had been associated with boscalid resistance.

We hypothesized that the exposure of boscalid-resistant strains of *B. jaapii* to fungicides containing fluopyram or fluxapyroxad would rapidly select for resistance to these compounds. However, we did not know if resistance evolution would occur sequentially, i.e., if *B. jaapii* strains containing the B-H260R mutation would evolve a second mutation conferring resistance to fluopyram and/or fluxapyroxad, or if resistance evolution would occur via selection of a new mutation in previously SDHI-sensitive strains. In this study, we tracked the evolution of resistance in field populations of *B. jaapii* at our test orchard at the Northwest Michigan Horticultural Research Center (NWMHRC) as fluopyram and fluxapyroxad lost CLS efficacy. We simultaneously performed a largescale orchard survey in Michigan of *B. jaapii* for resistance to fluopyram and fluxapyroxad, and assessed instances of practical resistance within individual orchard populations. Practical resistance of a target fungal pathogen occurs when reductions in the level of disease control are caused by the selection of fungicide-resistant isolates [19]. We further sequenced and identified mutations in the *SdhB* and *SdhC* genes of *B. jaapii* that were correlated with different resistance phenotypes to fluopyram and fluxapyroxad.

## 2. Materials and Methods

### 2.1. Field Efficacy of Fungicides Containing Fluopyram or Fluxapyroxad in Controlling CLS

Experimental field trials were conducted from 2017–2019 at the NWMHRC in Traverse City, MI, in a block of tart cherry cv. Montmorency trees (4 years old in 2017). Experiments were set up in a randomized complete block design, and we used single-tree plots with four replications per treatment. Each sprayed treatment consisted of six fungicide applications, made at 9–10 day intervals between petal fall and harvest (Table 1). In all of the experimental treatments excluding the unsprayed control, the broad-spectrum fungicide chlorothalonil was used for the first two applications. Chlorothalonil is the preferred fungicide for early-season CLS management [20], and is used by Michigan tart cherry growers with typical applications between the petal fall and shuck split phenological timings. Fungicide sprays were applied to individual trees to runoff using a portable hand-gun sprayer at a rate of 330 to 386 kg cm^−2^ and 458.9 L water ha^−1^. We relied on natural inoculum each year for infection of trees. Disease ratings were typically conducted at harvest and at approximately one month after harvest. We rated CLS disease incidence and defoliation on 20 randomly-selected terminal shoots per tree as previously described [4]. Data were analyzed using the analysis of variance and least significant difference mean comparison (*p* < 0.05) function of ARM (version 2020.0; Gylling Data Management Inc., Brookings, SD, USA). Percentage data were subjected to an acrsine square root transformation before analyses.

### 2.2. Orchard Sampling

During the growing seasons 2016–2019, leaves displaying symptoms of CLS were collected from 35, 39, 34, and 4 commercial orchards in Michigan, respectively, to generate mono-conidial isolates. The sampled commercial orchards were located in the major tart cherry-producing regions of Michigan, including several counties in the northwest (Antrim, Benzie, Grand Traverse, Leelanau, Manistee, and Mason), and west central (Kent and Oceana) areas of the state. Also, from 2016 to 2019, infected leaves were collected from unsprayed control trees from our research plot at the NWMHRC, where fungicide efficacy experiments were conducted. In the 2017 and 2018 surveys, CLS-symptomatic leaves were also collected from black cherry (*Prunus serotina*) trees located in a forest in Cuyahoga County, Ohio, and from a forest in Tuscola County, Michigan; both of these sites were not known to be sprayed with fungicides and were located far from commercial cherry orchards. The *B. jaapii* isolates recovered from these forests served as baseline isolates for determining fluopyram and fluxapyroxad sensitivity. An additional set of 30 commercial orchard isolates of *B. jaapii* from a 2003–2004 survey [2] was also tested in 2019. These were isolates that displayed reduced sensitivities to DMI fungicides in an earlier survey [2], but predated the use of SDHIs in commercial orchards.

Approximately 30 leaves were collected at random from 30 different cherry trees (1 leaf per tree) from each sampling site, placed in paper bags, and brought to the laboratory on ice. The leaves were stored at 5 °C prior to the isolation of conidia from acervuli on the infected leaves. To obtain mono-conidial isolates, one lesion per leaf was randomly selected, and conidia emerging from the lesion were streaked onto coffee agar medium (CWA; 20% brewed coffee, 2% agar). After 24 h, single germinated conidia were transferred and maintained on malt extract agar (MMEA; 2% malt extract, 0.1% yeast extract, 2% agar). Voucher specimens of each isolate were transferred to MMEA slants, which were maintained at 5 °C for long-term storage.

### 2.3. In Vitro Sensitivity Determination of B. jaapii to SDHI Fungicides

The minimum inhibitory concentration (MIC) method was used to evaluate the sensitivity of *B. jaapii* to the SDHI fungicides boscalid, fluopyram, and fluxapyroxad. The MIC method is the most appropriate method of evaluation for *B. jaapii* because of the very slow growth rate of the fungus of only a few millimeters of colony expansion over a 15-day time period [4]. The MIC method identifies the lowest concentration of fungicide that completely inhibits fungal growth. Fungicide stock solutions were prepared using the commercial formulation of Endura^®^ (BASF), which contains 70% a.i. boscalid, the commercial formulation of Luna Privilege^®^ (Bayer) that contains 41.5% of fluopyram, and the technical grade of fluxapyroxad (BASF). Stock solutions were prepared by dissolving the fungicides in acetone, which were later added to autoclaved, cooled glycerol yeast extract agar medium (GLYE; [21]), which contains 10 g of glycerol, 10 g of yeast extract, 6 g of NaNO_3_, 1.5 g of KH_2_PO_4_, 0.5 g of KCl, 0.5 g of MgSO_4_, and 15 g of agar in 1 L of water.

For the fungicide sensitivity screenings conducted in 2016 and 2017, a phenotypic sensitivity rating was assigned based on colony growth at the tested concentrations (0.01, 0.1, 0.5, 0, 2.5, 5, 10, 25, 35, and 40 µg mL^−1^ of active ingredient). For the 2018 and 2019 surveys, the fungicide concentrations tested were reduced to 0, 2.5 and either 25 µg mL^−1^ (boscalid) or 35 µg mL^−1^ (fluopyram and fluxapyroxad). The acetone concentrations were equalized for all of the treatments. Concentrations greater than 40 µg mL^−1^ a.i. were not assessed because of issues with precipitation of the studied fungicides in solution. Control medium, not amended with any fungicide, contained an equivalent amount of acetone as fungicide-amended medium. Mycelial plugs (1 mm) from actively-growing cultures on MMEA medium were transferred to the GLYE fungicide-amended media, and incubated for 15 days at 23 °C, after which the plates were examined for colony expansion, and the MIC for each isolate was determined as the minimum concentration at which the colonies failed to grow. A total of 1610 isolates from the 2016–2017 surveys were tested against all three SDHIs, and a total of 1088 isolates from the 2018–2019 surveys were tested against all three SDHIs. Isolates were categorized into three different phenotypic groups based on the in vitro assays in the 2016–17 surveys. Isolates with MICs ≤ 2.5 µg mL^−1^ were considered sensitive, isolates with MICs > 2.5 and ≤25 µg mL^−1^ were considered moderately resistant, and isolates with MICs ≥ 35 µg mL^−1^ were considered resistant to these fungicides. The same scale was used previously in our study of boscalid resistance in *B. jaapii* [4].

### 2.4. Amplification and Identification of Mutations within SdhB, SdhC, and SdhD of B. jaapii

We recently completed a draft genome sequence of *B. jaapii* that facilitated the identification of the *SdhB*, *SdhC*, and *SdhD* SDHI fungicide target genes [22]. Forward and reverse oligonucleotide primers were designed (Table 2) to facilitate the amplification of these gene sequences using the polymerase chain reaction (PCR). A total of 22 *B. jaapii* isolates with varying levels of sensitivity to boscalid, fluopyram, and fluxapyroxad were selected for analysis. DNA isolation from *B. jaapii*, PCR amplification, and purification of PCR fragments was done as described previously [4]. Purified PCR fragments were sequenced at the Michigan State University Genomics Technology Support Facility. To ensure the genes were fully sequenced on both strands and that we obtained accurate calls of the 5′ and the 3′ end sequences, we used both the initial primers used for amplification of the *B. jaapii SdhB*, *SdhC*, and *SdhD* genes, and additional sets of primers (SdhB_seq_F/ SdhB_seq_R, SdhC_seq_F/ SdhC_seq_R, and SdhD_seq_F/ SdhD_seq_R) to sequence the amplicons (Table 1). The sequencing chromatograms were analyzed using SnapGene^®^ software (from Insightful Science, San Diego, CA, USA; available at snapgene.com, accessed on 10 April 2021). Only calls passing the Phred quality score of 30 were used for gene assembly. The assembled gene and corresponding amino acid sequences were analyzed using Clustal Omega (https://www.ebi.ac.uk/Tools/msa/clustalo/, accessed on 10 April 2021).

## 3. Results

### 3.1. Field Efficacy of Fungicides Containing Fluopyram or Fluxapyroxad in Controlling CLS

We conducted experimental field trials at the NWMHRC from 2017–2019 to evaluate the efficacy of premix fungicides containing fluopyram (Luna Sensation, premix of fluopyram, and trifloxystrobin) and fluxapyroxad (Merivon, premix of fluxapyroxad, and pyraclostrobin), as well as fungicides containing either fluopyram or fluxapyroxad alone (Luna Privilege and Sercadis). Chlorothalonil was utilized for the first two applications for each treatment, as this is the grower standard fungicide used at these timings (petal fall and shuck split phenological stages). Where the SDHI active ingredient was tested alone, the rates utilized were equivalent to the rate of the material in the premix fungicides (Table 1). In 2017, defoliation due to CLS infection was significantly reduced in treatments with Luna Sensation or Merivon compared to the untreated control (Table 3). However, by 2018, the amount of defoliation in the Luna Sensation or Merivon treatments, although significantly reduced compared to the untreated control, was increased to commercially unacceptable levels (Table 2). In addition, defoliation levels in treatments utilizing the SDHI components alone were at 61.5 and 79.5% of untreated control levels for fluopyram and fluxapyroxad, respectively (Table 3). In 2019, the efficacy of the SDHI fungicides continued to erode, with rates of disease incidence equivalent to the untreated control for the premix fungicides and for fluopyram and fluxapyroxad alone by 8 August, and defoliation levels for fluopyram and fluxapyroxad alone were also not significantly different from the untreated control by 8 August (Table 3).

### 3.2. Orchard Sampling and In Vitro Fungicide Sensitivity Screening

A total of 876, 897, 957, and 132 isolates were collected in 2016–2019, respectively. In 2016, the collection consisted of 838 isolates from commercial orchards and 38 from the NWMHRC. In 2017, 843 isolates were obtained from commercial orchards, 34 isolates from the NWMHRC, and 20 isolates from the forest in Columbiana County, Ohio. In 2018, the collection consisted of 905 isolates from commercial orchards, 12 baseline isolates, and 40 from the NWMHRC. In 2019, 107 isolates were obtained from commercial orchards and 25 isolates from the NWMHRC. An additional set of 30 commercial orchard isolates of *B. jaapii* from a 2003–2004 survey was also tested in 2019. These were isolates that displayed reduced sensitivities to DMI fungicides in an earlier survey [2], but predated the use of SDHIs in commercial orchards.

**Figure 1:**
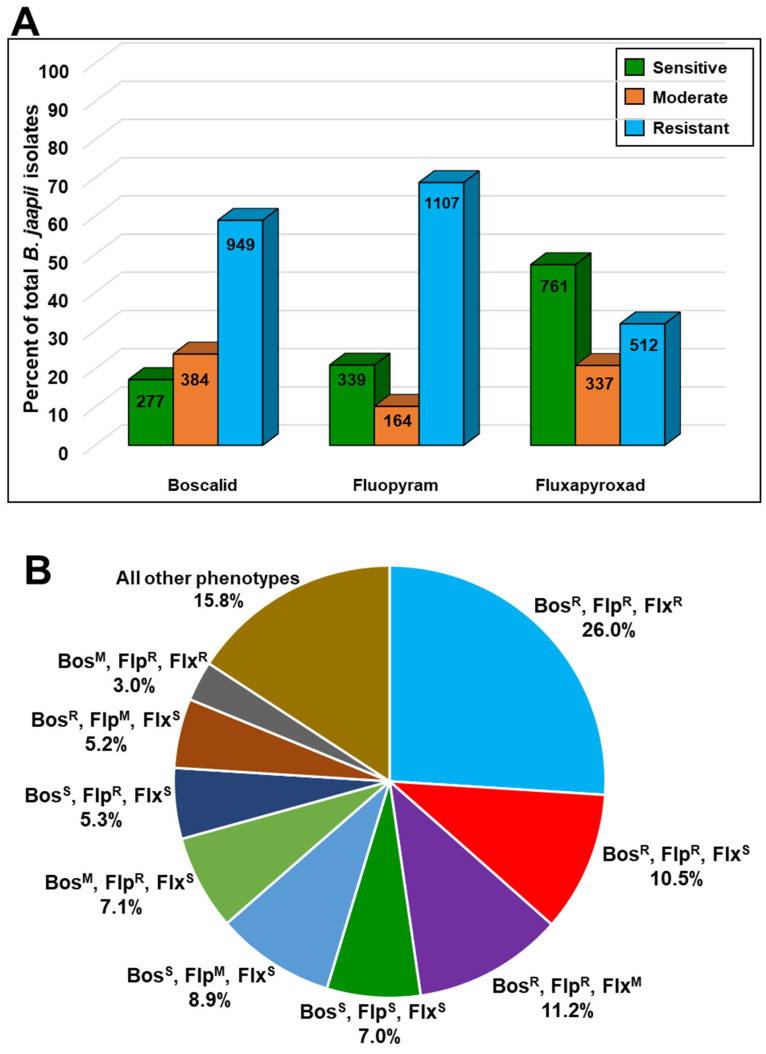
(**A**) Percentage of *Blumeriella jaapii* isolates rated as sensitive, moderately resistant, or resistant to boscalid, fluopyram, and fluxapyroxad from 2016–2017 surveys of commercial orchards in Michigan. (**B**) Percentages of *B. jaapii* isolates with various combined phenotypes of sensitive, moderately resistance, and resistant to boscalid, fluopyram, and fluxapyroxad from 2016–2017 surveys of commercial orchards in Michigan.

The overall percentage of sensitive, moderately resistant, and resistant isolates to boscalid, fluopyram, and fluxapyroxad for the 2016–2017 isolates is shown in Figure 1A. Resistance to boscalid (58.9%) and fluopyram (68.8%) was prevalent in the 2016–2017 isolate collection, while resistance to fluxapyroxad (33.9%) was much lower (Figure 1A). Likewise, the highest percentage of sensitive isolates in the 2016–2017 collection was to fluxapyroxad (47.3%). When considering combined phenotypes, a total of 26.0% of isolates were resistant to all three fungicides (Bosc^R^ Fluo^R^ Flux^R^; most common phenotypes) and only 7.0% were sensitive to all three fungicides (Bosc^S^ Fluo^S^ Flux^S^; Figure 1B and Table 4). The second and third most common phenotypes observed were Bosc^R^ Fluo^R^ Flux^M^ (11.2%) and Bosc^R^ Fluo^R^ Flux^S^ (10.5%) (Figure 1B and Table 4). Phenotypes in which only the fluopyram phenotype was altered from sensitivity (Bosc^S^ Fluo^R^ Flux^S^ and Bosc^S^ Fluo^M^ Flux^S^) accounted for 14.2% of the total (Figure 1B and Table 4). In contrast, we did not detect the Bosc^S^ Fluo^S^ Flux^R^ phenotype, and only 2 isolates (0.1%) exhibited the phenotype Bosc^S^ Fluo^S^ Flux^M^ (Table 4). Similarly, only 22 isolates (1.4%) had the phenotype Bosc^R^ Fluo^S^ Flux^S^ (Table 4).

The in vitro sensitivity to the three SDHIs of 30 isolates from commercial orchards collected in a 2003–2004 survey was also examined. These isolates had reduced sensitivity to DMI fungicides, but were collected prior to the use of SDHI fungicides. All 30 isolates were sensitive to boscalid and fluxapyroxad, and 15 isolates were sensitive to fluopyram with the other 15 isolates rated as moderately resistant.

### 3.3. Identification of Mutations within SdhB, SdhC, and SdhD of B. jaapii

We amplified and sequenced the *SdhB*, *SdhC*, and *SdhD* SDHI fungicide target genes from 22 *B. jaapii* isolates collected from commercial tart cherry orchards in the northwest and west central regions of Michigan. The phenotypes of the isolates were Bosc^R^ Fluo^S^ Flux^S^, Bosc^S^ Fluo^R^ Flux^S^, Bosc^S^ Fluo^R^ Flux^R^, and Bosc^R^ Fluo^R^ Flux^R^. Sequence analysis of three Bosc^R^ Fluo^S^ Flux^S^ isolates confirmed the presence of the B-H260R mutation detected in our previous study [4]; no mutations were detected in *SdhC* or *SdhD* in these isolates (Table 5). We detected a B-I262V mutation and a C-S84L mutation in two and six Bosc^S^ Fluo^R^ Flux^S^ isolates, respectively, and a C-N86S mutation in three isolates with the Bosc^S^ Fluo^R^ Flux^R^ phenotype (Table 5). Sequence analysis of eight isolates with the fully resistant Bosc^R^ Fluo^R^ Flux^R^ phenotype revealed three different genotypes: the C-N86S mutation alone in four isolates, the C-N86S mutation plus the B-I262V mutation in three isolates, and the C-S84L mutation plus the B-I262V mutation in one isolate (Table 5).

### 3.4. Assessment of Practical Fungicide Resistance in Michigan Tart Cherry Orchards

We first correlated the percentages of moderate and resistant *B. jaapii* isolates collected from unsprayed control trees in the NWMHRC fungicide efficacy research test plot with fungicide efficacy. The threshold for practical resistance was set for treatments where defoliation from CLS was greater than 50% in the August ratings. Between 2017 and 2019, the combined percentage of *B. jaapii* isolates that were either moderately-resistant or resistant to fluopyram was always above 97%, and the percentage of fluopyram resistance increased in each year (Table 6). The combined percentage of *B. jaapii* isolates that were either moderately resistant or resistant to fluxapyroxad was highest in 2018 and 2019 (both years > 82%), although the frequency of resistant isolates was relatively low (Table 6). The elevated frequency of moderately resistant and resistant isolates was associated with diminished efficacy of fluopyram and fluxapyroxad in this test orchard when used singly, and also when used in premixes with a QoI fungicide (Table 3). Practical resistance to fluopyram and fluxapyroxad was first observed in 2018, when the compounds were applied singly. In 2019, practical resistance was observed when the compounds were used singly, and also when used in premixes with a QoI fungicide (Table 3).

Based on the data from the fungicide efficacy research test plot, we defined practical resistance in commercial orchard populations in which the combined frequency of moderately resistant and resistant isolates was >97% for fluopyram and >82% for fluxapyroxad. In 2018 and 2019, *B. jaapii* isolates from commercial orchards were only tested at three fungicide concentrations to facilitate identification of each isolate as sensitive, moderately-resistant, or resistant. We screened isolate populations from 35 commercial orchards with a minimum of 17 isolates per orchard. Of the ten orchards located in west central Michigan, practical resistance to fluopyram and fluxapyroxad was detected in nine and seven orchards, respectively, and practical resistance to both fungicides occurred in seven orchards (Table 7). Of 25 orchards located in northwest Michigan, practical resistance to fluopyram and fluxapyroxad was detected in 20 and 7 orchards, respectively, and practical resistance to both fungicides occurred in 7 orchards (Table 7). In both regions, where practical resistance was only observed to one fungicide, that fungicide was always fluopyram.

## 4. Discussion

Our results show that resistance to boscalid, fluopyram, and fluxapyroxad in *B. jaapii* is widespread in commercial tart cherry orchards in the major growing regions of Michigan. Assessments of the phenotypic composition of resistant isolates from individual orchards has also shown that practical resistance to fluopyram and fluxapyroxad occurs in most of the commercial orchards surveyed. Although both fluopyram and fluxapyroxad are marketed in fungicide premixes with the QoI fungicides trifloxystrobin and pyraclostrobin, respectively, the efficacy of these fungicides is impacted by SDHI fungicide resistance (Table 3), even though resistance to QoIs has not been documented.

Assessment of a previous collection (2010–2011) of *B. jaapii* sampled from commercial tart cherry orchards in Michigan (similar locations to orchards sampled in this study) revealed that 30.4% of 1189 isolates were resistant to boscalid [4]. During this earlier time period, the collected isolates were sensitive to fluopyram and fluxapyroxad, and CLS was controlled with the premix fungicides Luna Sensation and Merivon [4]. Because of the resistance issue, the boscalid-containing premix fungicide Pristine (BASF) was replaced with Luna Sensation and Merivon in commercial tart cherry orchards in Michigan beginning in 2012. The B-H260R mutation was detected in all ten Bosc^R^ *B. jaapii* isolates examined in our previous study [4]. Because of the deployment of additional SDHI fungicides, and because the Bosc^R^ *B. jaapii* isolates all presumably contained a mutation that was not associated with fluopyram or fluxapyroxad resistance, we wondered if *B. jaapii* isolates from 2016–2017 that recently evolved resistance to fluopyram, fluxapyroxad, and also possibly to boscalid, would have evolved by acquiring additional mutations to B-H260R. To address this question, we sequenced the *SdhB*, *SdhC*, and *SdhD* genes from 19 *B. jaapii* isolates in an attempt to infer the subsequent evolution of SDHI resistance in Michigan populations of *B. jaapii*.

Two mutations, B-I262V and C-S84L, were detected among eight Bosc^S^ Fluo^R^ Flux^S^ isolates examined (Table 5). Homologous B-I229V and B-I280V mutations have been previously detected in fluopyram-resistant field isolates of *S. citrulli* and *C. cassiicola*, respectively [17,18]. The C-S84L mutation is a new fluopyram-resistance mutation. We hypothesize that both the B-I262V and C-S84L mutations evolved from SDHI-sensitive *B. jaapii* strains. In addition, both B-I262V and C-S84L mutations conferred fluopyram resistance following expression of the mutant alleles in a heterologous *Sclerotinia sclerotiorum* system [23].

Our results showed three genotypes among eight Bosc^R^ Fluo^R^ Flux^R^ isolates examined, none of which contained the B-H260R mutation (Table 5). Four of the isolates harbored a C-N86S mutation; this mutation has been detected in field populations of *Zymoseptoria tritici* in Europe [24], and the homologous C-N75S mutation in *SdhC* has been detected in field populations of *Pyrenophora teres* in Europe [25]. However, the C-N86S (C-N75S) mutation has generally been associated with low to moderate levels of resistance to SDHIs [24,26]. Likewise, we were not able to confirm the function of the C-N86S mutation in conferring resistance to fluopyram and fluxapyroxad following expression in the heterologous *S. sclerotiorum* system, but did find that this mutation confers resistance to two other SDHI compounds, pyraziflumid and inpyrfluxam [23]. The four Bosc^R^ Fluo^R^ Flux^R^ and three other Bosc^S^ Fluo^R^ Flux^R^ *B. jaapii* isolates harboring the C-N86S mutation displayed high levels of resistance to fluopyram and fluxapyroxad, but these isolates may have an additional mutation that was undetected. Three Bosc^R^ Fluo^R^ Flux^R^ isolates harbored the N86S mutation in *SdhC* with an additional B-I262V mutation, and one isolate had a combination of the B-I262V mutation along with a C-S84L mutation. While the B-I262V and C-S84L mutations were each individually correlated only with fluopyram resistance, it is possible that co-association of either of these mutations with the C-N86S mutation changes the fungicide-binding pocket resulting in additional fluxapyroxad resistance. We hypothesize that each of these Bosc^R^ Fluo^R^ Flux^R^ isolates evolved from an original genotype that was sensitive to all three SDHI fungicides.

**Figure 2:**
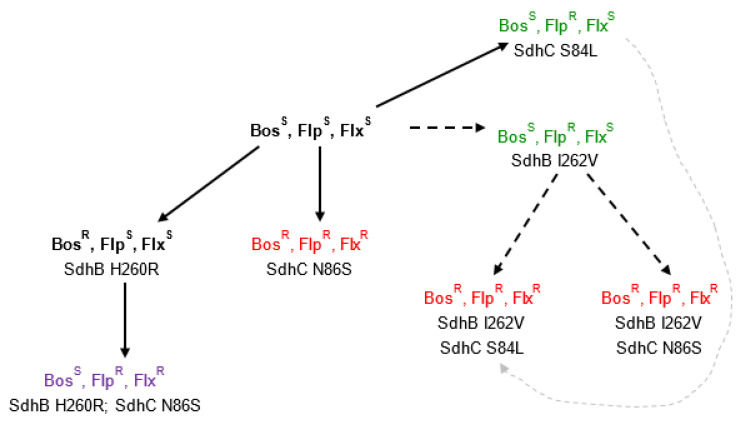
Evolutionary pathways to resistance to fluopyram and fluxapyroxad in *Blumeriella jaapii* based on known mutations occurring in the *SdhB* and *SdhC* genes of resistant strains. Strains with the Bosc^S^ Fluo^S^ Flux^S^ and Bosc^R^ Fluo^S^ Flux^S^ phenotypes are highlighted in bold and are the presumed progenitor phenotypes, since these strains were present in Michigan in 2010–2012 [4], prior to the registration of fungicides containing fluopyram or fluxapyroxad for commercial use. Strains with Bosc^S^ Fluo^R^ Flux^S^, Bosc^S^ Fluo^R^ Flux^R^, and Bosc^R^ Fluo^R^ Flux^R^ phenotypes are shown in green, purple, and red text, respectively. Filled lines represent direct emergence of mutations from original Bosc^S^ Fluo^S^ Flux^S^ and Bosc^R^ Fluo^S^ Flux^S^ backgrounds. Black dashed lines represent the putative order of evolution of resistant strains containing the B-I262V mutation. The gray dashed line indicates a possible alternative order of evolution of the Bosc^R^ Fluo^R^ Flux^R^ B-I262V + C-S84L genotype.

Thus, we hypothesize that there were a few evolutionary routes in the *B. jaapii* population ending in the fully-resistant Bosc^R^ Fluo^R^ Flux^R^ phenotype, and that all of these routes were initiated from a fully-sensitive Bosc^S^ Fluo^S^ Flux^S^ isolate (Figure 2). The first route involved the selection of the C-N86S mutation, and possibly an additional mutation(s) that we have not detected (Figure 2). The second route involved an intermediate Bosc^S^ Fluo^R^ Flux^S^ isolate harboring the B-I262V mutation in which second mutations, either C-S84L or C-N86S, were selected (Figure 2). An alternative evolutionary pathway to the Bosc^R^ Fluo^R^ Flux^R^ phenotype with B-I262V + C-S84L genotype would be via an intermediate Bosc^S^ Fluo^R^ Flux^S^ isolate harboring the C-S84L mutation in which the B-I262V mutation was subsequently selected (Figure 2). Older Bosc^R^ Fluo^S^ Flux^S^ strains in the population, prior to the use of Luna Sensation and Merivon in commercial orchards, could have given rise to Bosc^S^ Fluo^R^ Flux^R^ isolates through selection of the additional C-N86S mutation (Figure 2). The B-H260R + C-N86-S genotype was detected in one isolate, and we hypothesize that addition of the second mutation altered the structure of the SDHI-binding pocket resulting in the altered resistance phenotype.

It is interesting that resistance to three separate SDHI fungicides has evolved in *B. jaapii* populations in Michigan, even though each of these fungicides has been only applied in fungicide premixes, and the mixing partner QoI fungicide is effective in CLS management [4]. However, in the case of Luna Sensation, the preferred fungicide rate utilized by growers of 365.4 mL hectare^−1^ (5.0 fluid ounces per acre) contains only 91.4 g hectare^−1^ of trifloxstrobin, which is used at a rate of 136.1 g hectare^−1^ (3.8 fluid ounces per acre) when applied as the single fungicide Flint Extra (Bayer). This 49% reduction in amount of trifloxystrobin in the Luna Sensation premix likely resulted in a reduction in disease control by the trifloxystrobin component of the mixture, putting more selection pressure on the fluopyram component. The effect of dose rates, and the use of two single-site fungicides together in a mixture for fungicide resistance management have been studied in multiple pathosystems (ex. [27,28]), but optimal use strategies are clearly not universal. One idea emerging from the study of Ayer et al. [28] is that “managing population size may be one of the best strategies for reducing resistance development”. These authors conducted a four year field study on fungicide management of apple scab, caused by *Venturia inaequalis*. In this study, dose rate was important, as the authors noted a higher probability of reduced sensitivity or resistance emerging in populations exposed to lower doses of fungicides, including fluxapyroxad [28]. However, fungicide resistance to fluxapyroxad never evolved when this fungicide was used as part of a premix with various other fungicides [28]. While the apple scab and CLS diseases are similar in disease cycle [29,30], the successful management of apple scab early in the season can effectively eliminate this pathogen for the rest of the growing season [31]. In contrast, the incidence of CLS will continue to increase after harvest and into the fall, as it is simply not economically feasible to try to manage this pathogen throughout the entire growing season. This difference in pathogen population size may be a factor in the rapid evolution of SDHI resistance in *B. jaapii* in Michigan and the lack of SDHI resistance evolution in *V. inaequalis*, even though resistance to the QoI component of the SDHI premix fungicides Luna Sensation and Merivon is widespread in *V. inaequalis* [32,33].

## 5. Conclusions

In summary, we report the evolution of resistance to fluopyram and fluxapyroxad in populations of *B. jaapii* in Michigan, leading to practical resistance in a large majority of commercial tart cherry orchards. The evolution of fungicide resistance in *B. jaapii* to the most effective single-site fungicides for CLS disease has left growers relying on older, broad spectrum fungicides as previously predicted [34].

## Figures and Tables

**Table 1:** Fungicide spray programs utilized in cherry leaf spot disease control experiments conducted at the Northwest Michigan Horticultural Research Center, Traverse City, MI, USA, 2017 through 2019.

		Phenological Stages and Fungicides (g ha^−1^) Applied ^x^					
Treatment Description ^y^	Years	Petal Fall	Shuck Split	1st Cover	2nd Cover	3rd Cover	4th Cover
Control		--- ^z^	---	---	---	---	---
Fx + Py (P)	2017–2019	Ch 2780	Ch 2780	Fx 100 + Py 100	Fx 100 + Py 100	Fx 100 + Py 100	Fx 100 + Py 100
Fp + Tr (P)	2017–2019	Ch 2780	Ch 2780	Fp 92 + Tr 92	Fp 92 + Tr 92	Fp 92 + Tr 92	Fp 92 + Tr 92
Fx (P)	2018–2019	Ch 2780	Ch 2780	Fx 100	Fx 100	Fx 100	Fx 100
Fp (P)	2018–2019	Ch 2780	Ch 2780	Fp 92	Fp 92	Fp 92	Fp 92

^x^ Fungicide common names, brand names, formulations used, and manufacturer: Ch = chlorothalonil (Bravo Weather Stik 720SC, Syngenta Crop Protection, Inc., Greensboro, NC, USA); Fp = fluopyram (Luna Privilege, Bayer Crop Science LP, Research Triangle Park, NC, USA); Fx = fluxapyroxad (Sercadis, BASF Corp., Research Triangle Park, NC, USA); Fp + Tr = fluopyram + trifloxystrobin (Luna Sensation, Bayer Crop Science LP, Research Triangle Park, NC, USA); Fx + Py = fluxapyroxad + pyraclostrobin (Merivon 4.17SC, BASF Corp., Research Triangle Park, NC, USA). Numbers refer to amount of active ingredient applied (g ha^−1^). ^y^ Treatment description refers to compound and seasonal use, P = program. ^z^ --- indicates that no fungicide was applied corresponding to that phenological stage.

**Table 2:** Oligonucleotide primers used in this study. All primer sequences were newly designed in this study.

Purpose	Primer Name	Sequence (5′-3′)	Amplicon Size
Amplification and sequencing of *SdhB*	SdhB_amp_F	CCCAATAAGACACCTCAACTC	1094 bp
	SdhB_amp_R	ATAACACTCTCGCATCCCTA	
	SdhB_seq_F	GGTTGATCCGACGTTGAC	Not applicable
	SdhB_seq_R	GTACGGCTTGATGGACTTG	
Amplification and sequencing of *SdhC*	SdhC_amp_F	ATGTTGGCTCAACGAGCTG	1007 bp
	SdhC_amp_R	CTAATACGCCAATGCCAAGTAC	
	SdhC_seq_F	CTGCTGCCTTTGAGGATAG	Not applicable
	SdhC_seq_R	GCTTGTAGATGGAGAGGTTG	
Amplification and sequencing of *SdhD*	SdhD_amp_F	ATGGCATCAATTGTGCGAC	690 bp
	SdhD_amp_R	CTATGCCGCCCAGATCCT	
	SdhD_seq_F	TTCCCGGTTCCTTGAGAGT	Not applicable
	SdhD_seq_R	CCGTGGGAATGTAGTCGATTATG	

**Table 3:** Cherry leaf spot incidence and defoliation in field trial experiments conducted at the Northwest Michigan Horticulture Research Center, Traverse City, MI, USA.

		Cherry Leaf Spot Rating ^z^			
		Infection (%)		Defoliation (%)	
Year	Treatment Description ^y^	Harvest	Post-Harvest	Harvest	Post-Harvest
**2017**			**2 August**		**2 August**
	Fluxapyroxad + pyraclostrobin	--	37.3 c ^x^	--	6.7 b
	Fluopyram + trifloxystrobin	--	63.3 b	--	12.2 b
	Untreated control	--	88.6 a	--	89.6 a
**2018**		**20 July**	**30 August**	**20 July**	**30 August**
	Fluxapyroxad + pyraclostrobin	14.3 b	56.1 c	1.9 b	26.7 d
	Fluopyram + trifloxystrobin	21.5 ab	84.4 b	2.9 b	45.6 cd
	Fluxapyroxad	11.9 b	83.4 b	1.9 b	56.0 bc
	Fluopyram	25.2 ab	90.9 ab	5.7 b	72.4 b
	Untreated control	38.4 a	100.0 a	19.5 a	91.1 a
**2019**		**11 July**	**8 August**	**11 July**	**8 August**
	Fluxapyroxad + pyraclostrobin	55.7 bc	99.4 a	34.2 bc	59.2 b
	Fluopyram + trifloxystrobin	52.4 c	97.8 a	26.7 c	65.1 b
	Fluxapyroxad	70.5 ab	100.0 a	39.1 b	90.4 a
	Fluopyram	75.6 a	100.0 a	42.7 b	91.1 a
	Untreated control	74.6 a	100.0 a	57.2 a	96.9 a

^z^ Treatments were replicated four times on single-tree plots. Incidence was defined as percentage of leaves with CLS on 20 terminal shoots per tree. Percent defoliation was defined as (1 − [number of leaves/number of nodes]) × 100. ^y^ Treatment description refers to compound and seasonal use. For each treatment, the first two applications of the season were chlorothalonil, followed by four applications of the various treatments. Descriptions including fungicides and rates used correspond to those shown in Table 1. ^x^ Within an experiment, values followed by the same letter are not significantly different according to Fisher’s least significant difference test (*p* < 0.05).

**Table 4:** Frequency of occurrence of all possible combinations of phenotypic responses (sensitive [S], moderately resistant [M], resistant [R]) to the SDHI fungicides boscalid, fluopyram, and fluxapyroxad, of Michigan isolates (*n* = 1610) of *Blumeriella jaapii* collected in 2016 and 2017.

Fungicide Phenotype			Number of Isolates	Percentage of Isolates
Boscalid	Fluopyram	Fluxapyroxad		
S	S	S	112	7.0
R	R	R	418	26.0
S	R	S	85	5.3
S	S	R	0	0.0
R	S	S	22	1.4
R	R	S	169	10.5
R	S	R	5	0.4
S	R	R	15	0.9
M	M	M	15	0.9
S	M	S	144	8.9
S	S	M	2	0.1
M	S	S	9	0.6
S	M	M	6	0.4
M	M	S	23	1.4
R	M	S	83	5.2
R	S	M	14	0.9
R	M	M	42	2.6
M	R	M	63	3.9
M	M	R	5	0.3
S	M	R	5	0.3
S	R	M	15	0.9
M	R	S	114	7.1
M	S	R	0	0.0
M	R	R	48	3.0
R	M	R	16	1.0
R	R	M	180	11.2

**Table 5:** SDHI fungicide resistance profile and identified mutations in the succinate dehydrogenase genes of *B. jaapii* isolates from Michigan.

		Resistance/Sensitivity Phenotype			Presence/Absence of Mutation			
					*SdhB*		*SdhC*	
Isolate Name	Location	Bosc	Fluo	Fluxa	H260R	I262V	S84L	N86S
R0RB-17	Oceana	R	S	S	+			
VNSB-8	Oceana	R	S	S	+			
V2OB-12	Oceana	R	S	S	+			
ACBB-3	Leelanau	S	R	S		+		
LSTB-3	Mason	S	R	S		+		
ARPB-4	Leelanau	S	R	S			+	
WYOB-4	Oceana	S	R	S			+	
BMTB-1	Oceana	S	R	S			+	
JGFB-5	Mason	S	R	S			+	
NWSBC-2	Leelanau	S	R	S			+	
BMTB-5	Oceana	S	R	S			+	
GOOB-24	Leelanau	S	R	R				+
GSOB-6	Leelanau	S	R	R				+
WYOB-21	Oceana	S	R	R	+			+
ARPB-16	Leelanau	R	R	R				+
JGFB-3	Benzie	R	R	R				+
DRTB-4	Oceana	R	R	R				+
ACBB-21	Leelanau	R	R	R				+
DNFB-12	Leelanau	R	R	R		+		+
DCOB-26	Grand Trav.	R	R	R		+		+
JWNB-18	Oceana	R	R	R		+		+
LSTB-1	Mason	R	R	R		+	+	

**Table 6:** Number and percentage of isolates of *Blumeriella jaapii* from the Northwest Michigan Horticultural Research Center fungicide test plot that were either sensitive to, moderately resistant, or resistant to fluopyram and fluxapyroxad.

	Fluopyram Number (and Percentage) of Isolates			Fluxapyroxad Number (and Percentage) of Isolates		
Year	Sensitive	Moderate	Resistant	Sensitive	Moderate	Resistant
2017	0 (0.0)	21 (75.0)	7 (25.0)	13 (46.4)	10 (35.7)	5 (17.9)
2018	1 (2.5)	16 (39.5)	23 (57.5)	7 (17.5)	33 (82.5)	0 (0.0)
2019	0 (0.0)	9 (36.0)	16 (64.0)	4 (16.0)	18 (72.0)	3 (12.0)

**Table 7:** Assessment of practical resistance to fluopyram and fluxapyroxad in Michigan tart cherry orchards. The number (*n*) of *Blumeriella jaapii* strains isolated from each orchard is shown as well as the percentage of strains that were moderately resistant or resistant to each compound. Levels constituting practical resistance to either compound are highlighted in red ^a^. Orchards with practical resistance to fluopyram are highlighted in purple, and orchards with practical resistance to both fluopyram and fluxapyroxad are highlighted in red.

		Fluopyram		Fluxapyroxad	
Orchard ^b^	*n*	% Moderate	% Resistant	% Moderate	% Resistant
WC1	21	28.6	23.8	52.3	4.8
WC2	17	100	0.0	76.5	0.0
WC3	30	66.7	33.3	90.0	0.0
WC4	29	41.4	58.6	96.6	3.4
WC5	27	74.1	25.9	81.5	7.4
WC6	33	50.0	43.3	73.3	0.0
WC7	26	26.9	73.1	92.3	7.7
WC8	27	51.9	48.1	77.8	3.7
WC9	28	7.1	92.9	89.3	10.7
WC10	30	26.7	73.3	40.0	46.7
NW1	27	63.0	37.0	51.9	0.0
NW2	27	66.7	33.3	55.6	0.0
NW3	24	75.0	0.0	16.7	0.0
NW4	30	40.0	60.0	70.0	0.0
NW5	24	12.5	87.5	83.3	0.0
NW6	29	41.4	58.6	86.3	10.3
NW7	26	61.5	38.5	69.2	0.0
NW8	26	23.1	76.9	84.7	11.5
NW9	28	32.1	67.9	78.6	10.7
NW10	27	25.9	74.1	81.5	7.4
NW11	26	34.6	0.0	23.1	3.8
NW12	27	37.1	37.1	55.6	11.1
NW13	27	63.0	37.0	48.2	3.7
NW14	27	77.8	18.5	29.6	3.7
NW15	24	45.8	54.2	75.0	0.0
NW16	27	59.3	25.9	48.2	7.4
NW17	27	48.2	18.5	51.9	7.4
NW18	21	71.4	28.6	28.6	0.0
NW19	27	48.1	51.9	66.7	11.1
NW20	27	63.0	37.0	74.1	0.0
NW21	26	23.1	57.7	50.0	0.0
NW22	26	38.5	61.5	76.9	23.1
NW23	27	55.6	40.7	85.2	0.0
NW24	27	48.1	48.1	66.7	0.0
NW25	23	56.5	39.1	95.7	0.0

^a^ Practical resistance in orchards was determined as follows: Fluopyram, the percentage of resistant isolates within orchard is greater than 57% or the percentage of moderately-resistant plus resistant isolates is greater than 97%. Fluxapyroxad, the percentage of moderately-resistant plus resistant isolates is greater than 82%. ^b^ WC = west central region of Michigan, Kent and Oceana counties; NW = northwest region of Michigan, Antrim, Benzie, Grand Traverse, Leelanau, Manistee, and Mason counties.

## Data Availability

The complete *SdhB*, *SdhC*, and *SdhD* gene sequences from *B. jaapii* isolates reported in this study are deposited in GenBank under accession numbers MZ615182 (*SdhB*-I262V), MZ615183 (*SdhC*-WT), MZ615184 (*SdhC*-S84L), and MZ615185 (*SdhC*-N86S). The *SdhB*-WT and *SdhB*-H260R sequences were previously deposited in GenBank [4]. All other data presented in this study are available on request from the corresponding author.
